# Direct Germline Transformation of Cotton Meristem Explants With No Selection

**DOI:** 10.3389/fpls.2020.575283

**Published:** 2020-09-24

**Authors:** Yurong Chen, Andrea Lange, Zarir Vaghchhipawala, Xudong Ye, Annie Saltarikos

**Affiliations:** Plant Biotechnology, Bayer Crop Science, St. Louis, MO, United States

**Keywords:** cotton, *Gossypium hirsutum* L, germline transmission, meristem transformation, no selection, marker-free

## Abstract

Regeneration of transgenic plants without selectable markers can facilitate the development and commercialization of trait stacking products. A wide range of strategies have been developed to eliminate selectable markers to produce marker-free transgenic plants. The most widely used marker free approach is probably the *Agrobacterium*-based 2 T-DNA strategy where the gene-of-interest (GOI) and selectable marker gene are delivered from independent T-DNAs (Darbani et al., 2007). The selectable marker gene is segregated away from the GOI in subsequent generations. However, the efficiency of this 2 T-DNA system is much less than the traditional 1 T-DNA system due to the inefficiency of T-DNA co-transformation and high rate of con-integration between the GOI and selectable marker gene T-DNAs. In contrast, no selection transformation utilizes a single T-DNA carrying the GOI and thus eliminates the need to remove the selectable marker insert and potentially provides a viable alternative marker-free system. In this study, we reported the successful regeneration of transgenic cotton plants through *Agrobacterium* inoculation of seed meristem explants without the use of selective agents. Regeneration of putative transgenic plants were identified by GUS histo-chemical assay. The germline transmission of transgene to progeny was determined by segregation of pollen grains, immature embryos and T1 plants by GUS expression. The results were further confirmed by Southern analyses. The marker-free transformation frequency in this no selection system was similar to current meristem transformation system with selection (0.2%–0.7%). The strategy for further improvement of this system and its implication in improving cotton transformation pipeline and in developing transgene-free genome editing technology is discussed.

## Introduction

Plant transformation systems have evolved dramatically since the first successful reports in 1980s. Similar to other species, transgenic cotton plants were initially produced through somatic embryogenesis of hypocotyl or cotyledon cultures (Firoozababy et al., 1987; Umbeck et al., 1987) or suspension cell cultures (Finer and McMullen, 1990). These systems are tedious, laborious, genotype-dependent and have long plant regeneration cycles which result in tissue culture-induced low fertility of regenerated plants and other phenotypic changes (Li et al., 1989). To circumvent these problems, transformation of meristematic tissues through *Agrobacterium*, particle bombardment and other delivery methods followed by organogenesis was investigated in various academic and industrial laboratories. Cotton shoot apexes from 3–5 d old seedlings were initially proposed as an explant sources for transformation (Gould et al., 1991). However, the first successful protocol for *Agrobacterium*-mediated transformation of cotton shoot apex through kanamycin selection was only described several years later from the same laboratory (Zapata et al., 1999). This study demonstrated the stable integration of T-DNA into the cotton genome but the T0 transgenic tissues produced from this and subsequent studies in other laboratories were primarily epidermal and germline transformation was either not demonstrated or very low, under 0.3% (Keshamma et al., 2008; Gurusaravanan et al., 2020). In an independent study, embryonic axes from imbibed seeds were used for particle bombardment to produce putative transgenic events. However, the authors did not provide molecular data or demonstrate germline transmission through progeny analyses (Chlan et al., 1995). Stable transformation of embryonic axes by particle bombardment was reported through selection of a meristem-translocating herbicide, Imazapyr. An average germline transformation frequency of 0.55% was obtained in Brazilian cotton cultivars as demonstrated by molecular and progeny analyses (Aragao et al., 2005). The major breakthrough of cotton transformation technology was the development of *Agrobacterium-*mediated transformation of cotton meristem tissues of imbibed mature seeds using spectinomycin selection. This meristem transformation system was rapid, high throughput and genotype flexible (Chen et al., 2014). Successful transformation was obtained from three genotypes with a diverse genetic background. Molecular and progeny analyses demonstrated stable integration and expected Mendelian inheritance in most of the events (Chen et al., 2014). It was concluded that tissue culture parameter optimization and *Agrobacterium* improvement contributed to the development of genotype flexible and high throughput meristem transformation systems (Chen et al., 2014; Ye et al., 2016).

As demonstrated, these traditional plant transformation systems require a selection step for the production of stable transformed cells regardless of regeneration through embryogenesis of callus cultures or organogenesis of meristem explants. The small portion of transformed cells can be selected in the medium containing a selective agent through the expression of selectable marker genes against an overwhelmingly large percentage of non-transformed cells. Transgenic plants containing gene of interest (GOI) linked to, or independently inserted into the chromosome of the same cell as the selectable marker (SM) are regenerated from the transformed cells with visually identifiable phenotype. However, the presence of selectable markers in transgenic plants could hinder trait stacking efforts through retransformation given that the number of selectable markers suitable for each species is usually very limited (Ramana Rao et al., 2011).

A wide range of strategies have been proposed and developed to eliminate selectable markers to produce marker-free transgenic plants (Darbani et al., 2007; Yau and Stewart, 2013). Some of the most studied strategies are co-transformation of GOI and selectable marker gene followed by segregation of selectable marker gene in the next generation (Komari et al., 1996; Chen et al., 2014), transposon-mediated reposition (Goldsborough et al., 1993), site-specific recombinase-mediated selectable marker gene excision (Dale and Ow, 1991), homologous recombination (Puchta, 2002). The most widely used marker free strategy is probably the co-transformation strategy-2 T-DNA strategy where GOI and selectable marker gene are located in two separate T-DNA regions within the same plasmid. However, transformation frequency in this co-transformation system is much less efficient than traditional 1 T-DNA system due to the efficiency of co-transformation and linkage of GOI and selectable marker gene (Afolabi et al., 2004; Radchuk et al., 2005). In addition, marker-free events can only be recovered in T1 generation through sexual propagation.

Regeneration and identification of marker free transgenic plants without selection could avoid the problem of low co-transformation efficiency and high frequency of linkage of GOI and selectable marker gene encountered with the co-transformation strategy. The unlinked frequency of GOI and selectable makers for 2 T-DNA system in cotton is under 10% (Chen, unpublished observations). Furthermore, development of an efficient no selection transformation system would provide opportunity to enable transgene-free (Ribonucleoprotein-RNP) genome editing (Woo et al., 2015; Svitashev et al., 2016). Regeneration of transgenic plants without selection was initially reported in potato (De Vetten et al., 2003). The transformation frequency was reduced approximately by half compared to the selection approach based on PCR analysis of T0 plants (Kim et al., 2007). However, in both cases, no progeny analyses to determine germline transmission frequency were attempted. In barley, a transformation frequency of 0.8% was obtained without selection and 3.1% with hygromycin selection in *Agrobacterium-*mediated transformation (Holm et al., 2006). More recently, transformation frequency of 1.1%–3.1% was obtained in tobacco without selection whereas transformation frequency of over 90% was achieved when kanamycin selection was used. Progeny analyses showed that 28%–56% of events were chimeric or escape but no Southern analyses were conducted (Li et al., 2009). In all these no selection experiments, transgenic shoots were produced through leaf disc or callus phase. The successful transformation of cotton meristems through particle bombardment of excised embryonic axes with no selection was reported previously (McCabe and Martinell, 1993). But germline transformation frequency of 0.03 to 0.22% or 0.0%–0.04% (Keller et al., 1997) was obtained only after many rounds of tedious selective pruning of non-transformed primary shoot tips. In this study, we report direct regeneration of germline transgenic cotton plants from *Agrobacterium tumefaciens-* mediated meristem transformation with no selection with an average germline transformation frequency of 0.2%–0.7%.

## Materials and Methods

### Plant Materials and the Transformation Vector

**Figure 1:**
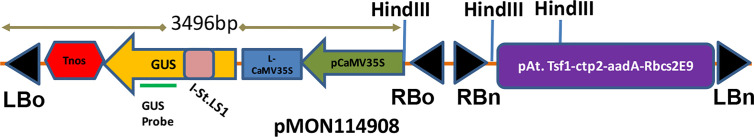
T-DNA structure of pMON114908. LBo/RBo: Left/Right T-DNA border from *Agrobacterium* octopine strain; LBn/RBn: Left/Right T-DNA border from *Agrobacterium* nopaline strain; P-CaMV 35S: enhanced cauliflower mosaic virus 35S RNA promoter; GUS: β-glucuronidase; Tnos: nopaline synthase terminator; P-At.Tsf1: *Arabidopsis* transcription factor 1 promoter; *ctp2*: chloroplast transit peptide 2; *aadA*: spectinomycin resistance gene; Rbcs2E9: pea rbcs2 E9 transcription terminator.

Cotton cultivar DP 393 was used in this study. Meristem explants were produced as described previously (Chen et al., 2014). Briefly, delinted cotton seeds were surface sterilized with 10% Clorox (0.615% sodium hypochlorite) for 10 min and rinsed with sterile water 4 times. Meristem explants were excised from the surface sterilized seeds following imbibition at 25°C for 16–22 h and purified by floatation in water. A disarmed *Agrobacterium* *tumefaciens* strain AB33-a VirG I77V mutant derivative, conferring a hypersensitive reaction to induction signals and derived from a nopaline type strain, ABI was used. The generation of this mutant strain and the underlying molecular mechanism of AB33 in improving cotton meristem transformation was previously described (Ye et al., 2016). The binary vector pMON114908 contains two independent T-DNAs (2-T) having the gene of interest (GOI), an intron-disrupted *uidA* encoding GUS (β-glucuronidase) within one T-DNA and the selectable maker, *aadA* gene within the 2^nd^ T-DNA border. The schematic representation of this vector is shown in **Figure 1**. Digestion of genomic DNA of transgenic plants with HindIII would generate a fragment that is equal to or larger than 3.5 kb with the GUS probe during Southern analysis. This combination will also allow for an estimation of copy numbers of inserted DNA. The same 2 T-DNA vector was used to develop initial cotton meristem transformation system with spectinomycin selection (Chen et al., 2014) and is used in this study with no selection for the comparison.

### *Agrobacterium*-Mediated Transformation Process

The preparation of *Agrobacterium* inoculum and co-culture of meristem explants with *Agrobacteium* were conducted as described previously (Chen et al., 2014). Briefly, overnight-grown *Agrobacterium* culture broth was centrifuged at 3500 rpm at 4°C for 25 min and then re-suspended in standard inoculation medium at O.D._660_ of 0.3. The standard inoculation medium consisted of 2/5 macro salts, 1/10 micro salt and 1/10 vitamins of Gamborg’s original B_5_ medium (Gamborg et al., 1968) supplemented with 30 g/L glucose, 3.9 g/L MES and with final pH of 5.4.

Fresh meristem explants (12–24 g) were submerged in prepared *Agrobacterium* inoculum a Plantcon (MP Biomedicals) and then subjected to sonication at 45 kHZ for 2 min in an ultrasonic cleaner (Sanpa W-113) and shaking at 80 rpm for 10 min. Inoculated explants were then transferred to co-culture medium in Plantcon with 2 ml of liquid co-culture medium. The liquid co-culture medium was the same as the inoculation medium but supplemented with 50 mg/L Nystatin and 10 mg/L thiabendazole (TBZ) for controlling any potential fungal growth. Co-culture was conducted in Percival incubators with the temperature of 23°C, relative humidity of 70% and a photoperiod of 16-light and 8-h dark at an intensity of 90 µmoles/m^2^/s for 3 days.

Co-cultured explants were transferred to the optimized shoot regeneration media and carried out as recommended previously (Chen et al., 2014). The regeneration medium consisted of salts and vitamins of Gamborg B5 (Phytotechnology G396), 1.29 g/L calcium gluconate, 20 g/L dextrose, 200 mg/L carbenicillin, 200 mg/L cefotaxime, 100 mg/L Timentin but without the selectable agent, spectinomycin. The medium was solidified with 4g/L agargel. Approximately 0.5 g of explants were surface-plated on the medium in each Plantcon. The cultures were placed in a tissue culture room at 35°C for 3 days before moved to 28°C. All the tissue culture rooms had a photoperiod of 16-light and 8-h dark at an intensity of 100 µmoles/m^2^/s. Explants with continuously growing green shoots were transplanted to oasis plugs (Jiffy Product of America, Inc.) initially and then transplanted to large soil pots for seed production after they were confirmed transgenic by histochemical assay for GUS. For GUS assay, plant materials were submerged in a 5-bromo-4-chloro-3-indolyl-β-glucuronic acid solution as previously described and incubated at 37°C for 6 h to overnight (Jefferson et al., 1987).

### Analysis of T0 Transgenic Events

Leaf tissues of green shoots in oasis soil plugs were sampled for GUS histo-chemical assay. Transformation frequency (TF) was defined as the percentage of GUS positive plants based on the number of events assayed. Transgenic plants identified by leaf GUS assay were transplanted to 6” diameters soil pots for further development. For some events, pollen grains from flowers were collected and/or developing immature embryos were dissected out from bolls for GUS histo-chemical assay to get an early read of germline transgene transmission. Seeds were harvested from selected events for progeny analyses.

### Analyses of T1 Progenies

Forty-eight T1 seeds harvested from putatively transformed T0 plants were germinated. Leaf tissues from 2-week old seedlings were collected for GUS assay. Progeny segregation data were analyzed by Chi-square test to determine the pattern of transgene transmission and the number of functional gene loci.

A sub-set of samples were analyzed through Southern analyses. For Southern analyses, genomic DNA from leaf samples of GUS positive T1 plants was extracted as previously described (Dellaporta et al., 1983). DNA was resuspended in 0.1X TE (pH8.0) buffer and treated with RNAse (10mg/ml stock) before storage at -20°C. DNA concentrations were determined with a NanoDrop Spectrophotometer (Thermo Fisher). Approximately 15–20 μg of DNA was digested overnight at 37°C in a 100 μl volume with HindIII (NEB) followed by alcohol precipitation and resuspension in 25 μl of milliQ water (Promega). Digested DNA from non-transgenic plants and T1 GUS positive plants was separated on a 1% agarose gel along with a positive control vector. DIG-labelled λ-HindIII digest (Roche) was run alongside the digests as a molecular size marker. Gels were run overnight at 35V and processed for southern blotting according to standard molecular biology protocols (Sambrook et al., 1989). DNA bound to nylon membranes (Hybond-N, GE healthcare) was crosslinked in a UV-Crosslinker (Stratagene). DIG labeled GUS probes were generated by PCR (PCR DIG-labelling Kit, Roche) and PCR products were gel purified and resupended in TE buffer. Membranes were hybridized with GUS probe labeled with DIG, washed and reacted with anti-DIG antibody conjugated with Alkaline Peroxidase according to protocol (Roche). Following antibody binding, a chemiluminescent AP substrate CSPD (Roche) was added for signal development which was recorded on X-ray film (Kodak Biomax).

## Results

### Regeneration and Identification of Putatively Transformed Cotton Plants Derived From Transformation of Meristem Explants With No Selection

**Figure 2:**
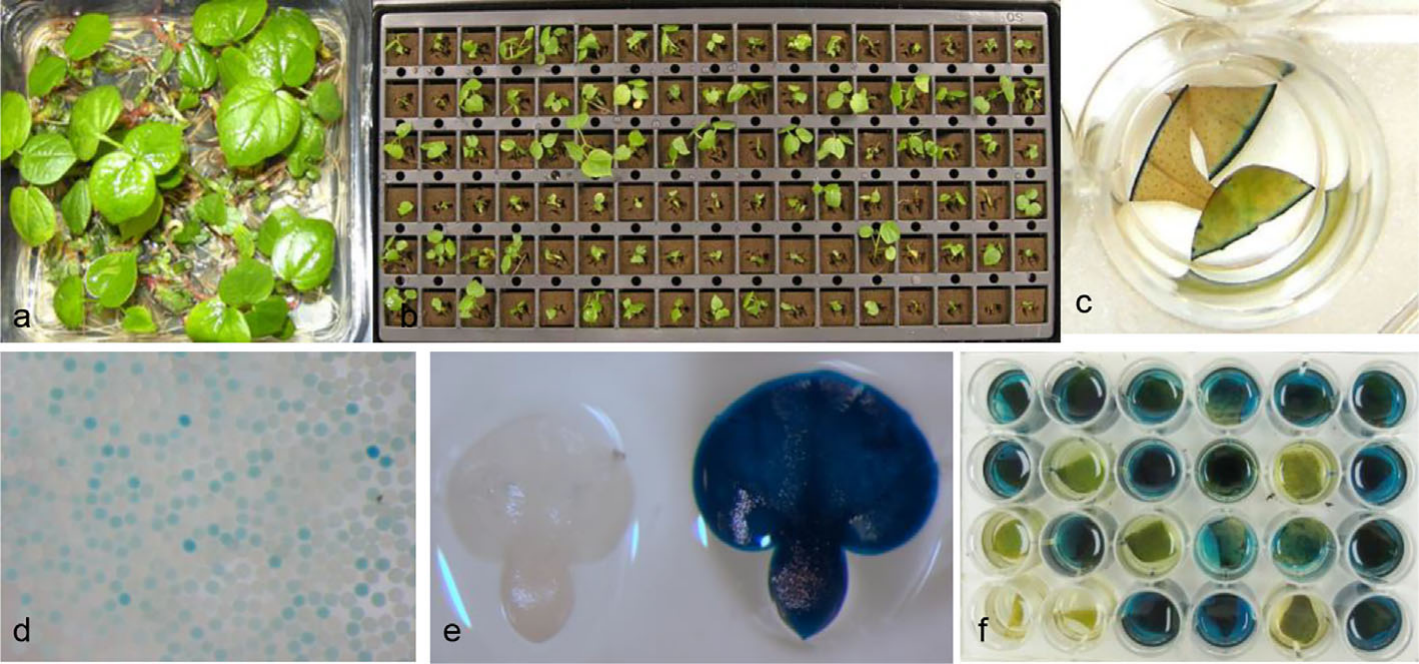
Regeneration and identification of putative transgenic cotton plants with no selection. **(A)** Regeneration of shoots from *Agrobacterium* co-cultured meristem explants after about 3 weeks culture on shoot regeneration medium with no selection; **(B)** Growth of regenerated plants in 6 x 17 formatted oasis plug; **(C)** Further confirmation of GUS positive plants by staining of three newly developed leaves from a single initially identified positive leaf; **(D)** Segregation of GUS positive and negative pollen grains; **(E)** Segregation of GUS positive and negative immature embryos of T1 seeds; **(F)** Segregation of GUS positive and negative T1 plants.

In a typical cotton meristem transformation protocol with selection, explants after co-culture were transferred to shoot regeneration medium with appropriate levels of spectinomycin. After selection for 4–5 weeks, apical meristem cells were inhibited or bleached and axillary buds in meristematic regions developed into small shoots. They were subsequently transferred to fresh regeneration medium for further development and root formation before being transplanted to soil pots (Chen et al., 2014). In no selection experiments, shoots were regenerated earlier and grew faster than in the experiments with selection. Lots of green shoots were regenerated with extensive root formation on regeneration medium after culture for only 3 weeks (**Figure 2A**). To facilitate the sampling and identification of putatively transformed T0 plants, the regenerated shoots in Plantcons were transplanted in 6 x 17 well formatted oasis plug (**Figure 2B**). Leaf tissue samples from the same row or column were pooled for GUS assay to reduce the handling of high percentage of escapes in no selection experiments. Individual GUS positive plants were subsequently identified in the pools with GUS positive leaf tissue. After an additional 2 to 3 weeks, newly developed leaves (usually 3) were sampled for further confirmation of the initial GUS positive plants (**Figure 2C**). Putatively transformed plants derived from no selection were phenotypically normal in greenhouse and had a clear segregation of pollen grains (**Figure 2D**) and immature embryos (**Figure 2E**) after GUS expression assay. From a total of 4,667 shoots regenerated from 7 independent experiments, the average putative TF based on initially number of regenerated shoots was 1.1% with the highest being 2.2% (**Table 1**).

**Table 1:** Transformation frequency of cotton meristem transformation with no selection based on GUS assay.

Exp #	# of shoots assayed	# GUS ^+^ plants	% TF
Exp 2458	312	7	2.2
Exp 2561	561	4	0.7
Exp 2585	586	4	0.7
Exp 2602	532	7	1.3
Exp 2588	862	13	1.5
Exp 2601	872	4	0.5
Exp 2604	942	11	1.2
Summary	4667	50	1.1

### Confirmation of Direct Regeneration of Germline Transgenic Plants Through T1 Analysis and Molecular Characterization

T1 seeds were harvested from 31 events regenerated from 7 independent experiments. T1 plants were analyzed by GUS assay to determine the transmission and segregation pattern. Ten of 31 (32.3%) events had germline transmission while the remaining 21 events did not produce any GUS positive progeny and were therefore considered as epidermal transformation. For 10 germline transformed events, some of them had clear GUS positive and negative segregation (**Figure 2F**). Fifty percent of germline events (5 out of 10), Event A_71040, A_71045, A_71046, A_73473, and A_84993 had a single locus of functional gene integration. Two events A_73361 and A_73412 had two functional loci of transgene integration whereas another two events, A_51949 and A_73360 did not have GUS negative plant in all 48 T1 progeny analyzed, indicating that 2 or more loci of functional transgene were integrated into cotton genome and the population was too small to recover GUS negative segregants. Event A_70929 produced fewer GUS positive T1 plants than expected and was considered as a chimera (**Table 2**).

**Table 2:** Segregation analyses of GUS assay in T1 progeny.

Event #	Estimated T0 copy #*	T1 GUS assay		Goodness of fit**			GUS functional locus
		positive (+)	Negative (-)	Chi-square (1:1)	Chi-square (3:1)	Chi-square (15:1)	
A_51949	9	48	0	46.02	14.69	2.22	≥2
A_70929	8	19	28	1.36	28.15	219.08	chimera
A_71040	3	36	10	13.59	0.12	16.28	1
A_71045	3	33	15	6.02	0.69	47.02	1
A_71046	2	34	12	9.59	0.00	27.60	1
A_73360	3	48	0	46.02	14.69	2.22	≥2
A_73361	5	40	3	30.14	6.52	0.01	2
A_73412	10	46	2	38.52	10.03	0.09	2
A_73473	3	35	12	10.30	0.01	26.62	1
A_84993	10	33	12	8.89	0.01	28.62	1

*Estimated T0 copy # was based on Southern analysis.

**Chi-square value < 3.84 indicates that the observed ratio fits the expected ratio with 95% confidence.

**Figure 3:**
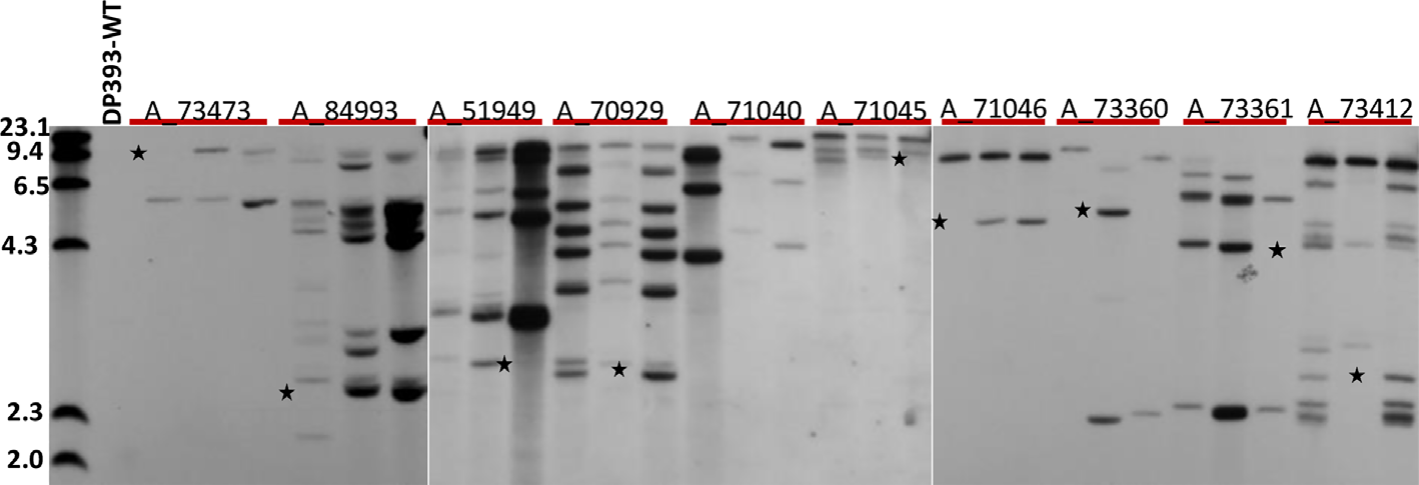
Southern analysis of 30 T1 plants from 10 T0 events. Genomic DNA from 30 T1 progenies of 10 T0 events (3 plants from each T0 event) and non-transformed (DP393 wild type) were digested with Hind III and probed with GUS. The first lane was molecular size marker. * indicates bands that segregates in three progeny plants of each T0 event tested.

Three T1 GUS positive plants from each of 10 T0 events were used for Southern analyses (**Figure 3**). Southern analyses showed all T0 events had intact GUS cassettes as each of them had at least one fragment larger than 3.5kb in size. Four events, A_71040, A_71045, A_71046, and A_73473, only had intact GUS fragments. The remaining 6 events, A_51949, A_73360, A_73361, A_73412, A_70929, and A_84993 also had truncated GUS fragments along with intact GUS fragments. Nine out of 10 events (90%), i.e., A_51949, A_70929, A_71045, A_71046, A_73360, A_73361, A_73412, A_73473, and A_84993 showed clear segregation of genomic fragments in 3 T1 plants. One of them, A_71040 did not have segregation since all three T1 plants had three intact GUS fragments. This may indicate that three intact GUS fragments were tightly linked in the cotton genome and inherited as a single locus. Alternatively, the population size was too small to recover other segregants. Interestingly, no single copy events were found in this study (**Figure 3**).

Most T0 events (four out of 5) with a single functional locus in segregation study, A_71040, A_71045, A_71046, and A_73473 had a low copy number (2 to 3) of GUS fragments. But one event A_84993 that had estimated 10 copies of GUS fragments also showed a single functional locus in the segregation study. This may indicate that all fragments within A_84993 were integrated into a tightly linked genomic region. Alternatively, this was most likely caused by physical truncation of some GUS fragments. On the other hand, all four T0 events with 2 or more single functional loci in segregation study, A_51949, A_73360, A_73361, and A_73412 had a high copy number (3 to 10) of GUS fragment. This indicates that multiple intact GUS fragments were independently inserted into different genomic regions and inherited as independent functional loci. T0 event A_70929 was considered as a chimeric due to fewer than expected GUS positive T1 plants in segregation study but it had estimated 8 copies of GUS fragments. The chimeric nature of this events only allowed partial germline transmission of the intact GUS fragment. Alternatively, the aberrant segregation in this event could be due to gene silencing or insertional mutation leading to the reduced transmission of transgene through male or female gametes.

## Discussion

To our knowledge, this is the first demonstration of *Agrobacterium* mediated direct germline transformation of meristem explants through organogenesis without selection. Base on an average of 32.3% germline transmission and the range of 0.5%–2.2% T0 putative transformation frequency, the marker-free germline transformation frequency in this experiment would be in the range of 0.16%–0.71%. In co-transformation experiments with 2 T-DNA vectors, usually less than 10% of events have independent insertion of GOI and selectable marker gene where marker free events could be generated in T1 generation. Therefore, the marker free TF demonstrated in this no selection study would be equivalent to 2%–8% TF in the experiments with traditional selection. The advantage of no selection is to save one generation to obtain marker free events compared to 2 T-DNA system where marker free events can only be recovered at the T1 generation. Even though we only describe the direct regeneration of maker free germline events in T0 generation with GUS as a GOI in detail in this study, the transformation procedure presented here can be easily applied for other trait genes, such as an insect resistance gene or a herbicide resistance gene. Therefore, this no selection transformation system can potentially help speed up the development and commercialization of trait stacking products in the cotton biotech pipeline. Furthermore, this cotton meristem transformation system with no selection is highly likely genotype flexible since we demonstrated similar transformation frequency in three genotypes with a diverse genetic background with spectinomycin selection in our previous studies with the same construct and under similar tissue culture conditions (Chen et al., 2014).

Our previous study showed that it took 8–9 weeks from initial explant inoculation to plugging of transgenic plants to soil plug in a typical cotton meristem transformation protocol with selection (Chen et al., 2014). In no selection experiments, transgenic shoots were regenerated earlier and grew faster than in the experiments with selection and can be plugged within 4-weeks following inoculation. However, heritable transformation frequency of 0.2%–0.7% was low in no selection experiments compared to 2%–8% in selection experiments in production. In addition, about 67% of T0 events in this no selection experiments were found to be epidermal and did not transmit any transgenes to the next generation. Reduced transformation frequency and germline transmission due to high frequency of epidermal transformation and/or high degree of chimerism were observed in both somatic embryogenesis-based transformation system and meristem-based organogenesis system in experiments with no selection compared with selection (McCabe and Martinell, 1993; Li et al., 2009). The reduction of transformation frequency could be explained by competitive disadvantage of very limited numbers of transformed cells as they are surrounded by an overwhelmingly larger number of non-transformed cells competing for growth. In addition, the limited number of transformed cells may have physiological growth disadvantage due to metabolic burden by expressing additional genes (Khan and Maliga, 1999). High frequency of epidermal transformation could be mainly explained by lack of the selection during regeneration. In 10 of T0 events that showed unambiguous evidence of germline transmission of transgene to the next generation in this no selection study, 70% of them followed a Mendelian segregation of 1 or 2 functional loci. Interestingly, Southern analysis did not identify any event with a single copy of transgene. This is a bit surprising since usually about 30% of single copy events are produced in cotton meristem transformation with spectinomycin selection in production (Chen, unpublished). A plausible explanation is that the events expressed at a low level or silenced due to complex T-DNA insertion have been eliminated when selection agent is used. But all events are recovered irrespective of gene expression level when there is no selection. This could also be due to the small sample size used for analysis in this study. Analysis of a large number of germline events could help to better understand the distribution of copy number with no selection transformation.

As hypothesized and demonstrated in the literature, transformation of a single cell in the original meristematic tissue of an embryonic axis could produce all germline tissue for the whole plant (Sussex, 1989; Irish, 1991). Hormonal manipulation could cause the formation of clonal sectors from which multiple shoots were regenerated through the organogenesis pathway. Furthermore, the transformation process, either particle bombardment or pre-treatment, such as sonication during *Agrobacterium*-mediated transformation could exert some level of damage to the meristem and cause reprogramming of individual cells in meristematic layers. The degree of chimerism of each shoot would depend on the number of surviving meristematic cells within each of three physiological layers, L1, L2, and L3. L1 layer is responsible for epidermis whereas L2 and L3 layers give rise to mesophyll and germline cells and vascular tissue of the plants respectively (Sussex, 1989). Therefore, further optimization of hormone type and level in tissue culture medium and physical, chemical, and biological wounding of apical meristem regions to influence the reprogramming of meristematic cells would potentially further help increase the transformation frequency and reduce chimerism frequency in transformation.

The successful germline transformation of cotton meristem through particle bombardment of excised embryonic axes from imbibed seeds with no selection was reported previously (McCabe and Martinell, 1993). The recovery of germline plants was tedious, labor intensive and through many rounds of selective pruning of non-transformed primary shoot tips and forcing the buds in the axils of transformed leaves to develop. Germline transformation frequency varied from genotype to genotype, ranging from 0.03% to 0.2% or 0.0%–0.04% (Keller et al., 1997) which was much lower than 0.16%–0.71% obtained in this study. The same type of explants was used in these two previous reports and our study. The difference observed was probably due to the delivery method of *Agrobacterium* versus particle bombardment and/or tissue culture medium and plant regeneration protocol. Application of explant wounding methods, such as sonication, tissue culture media and other learnings gained in this study may help the development of protocol for direct regeneration of transgenic plants from meristem explants through particle bombardment. Evidently, this and further optimized no selection system could help enable transgene-free (RNP-based) genome editing technology.

One of the major bottlenecks in no selection transformation is the need to screen large number of plants in order to find the few transformed plants. This is tedious and laborious. Development and application of simple, rapid, low cost, and point-of-care molecular tests such as recombinase polymerase amplification (RPA) or specific high-sensitivity enzymatic reporter unlocking (SHERLOCK) could potentially help to solve this problem (Piepenburg et al., 2006; Kellner et al., 2019).

In conclusion, we demonstrated successful germline transformation of cotton meristem with no selection. Direct regeneration of marker-free transgenic plants can eliminate the additional generation to remove selectable marker in traditional transformation protocol. Therefore, no selection transformation system can subsequently speed up the development and commercialization of trait stacking products in cotton biotech pipeline. Furthermore, development of a rapid and genotype flexible meristem transformation system with no selection could help enable transgene-free, RNP delivery-based genome editing technology in diverse germplasm of cotton. We anticipate that learnings gained from this study would be valuable to other dicots or meristem transformation systems in developing no selection protocols.

## Data Availability Statement

All datasets presented in this study are included in the article/supplementary material.

## Author Contributions

YC and AL conceived, designed, and coordinated the study. ZV and XY conducted molecular analyses. YC and AS drafted the manuscript.

## Conflict of Interest

All authors were employed by Bayer Crop Science at the time of research.
